# Safety and immune responses after intradermal application of Porcilis PRRS in either the neck or the perianal region

**DOI:** 10.1371/journal.pone.0203560

**Published:** 2018-09-07

**Authors:** Julia Stadler, Lena Naderer, Lisa Beffort, Mathias Ritzmann, Daniela Emrich, Walter Hermanns, Kerstin Fiebig, Armin Saalmüller, Wilhelm Gerner, Bernadette Glatthaar-Saalmüller, Andrea Ladinig

**Affiliations:** 1 Clinic for Swine, Centre for Clinical Veterinary Medicine, Ludwig-Maximilians-University Munich, Oberschleissheim, Germany; 2 Institute of Veterinary Pathology, Centre for Clinical Veterinary Medicine, Ludwig-Maximilians-University Munich, Munich, Germany; 3 MSD Animal Health, Unterschleissheim, Germany; 4 Institute of Immunology, Department of Pathobiology, University of Veterinary Medicine Vienna, Austria; 5 Labor Dr. Glatthaar, Ochsenhausen, Germany; 6 University Clinic for Swine, Department for Farm Animals and Veterinary Public Health, University of Veterinary Medicine Vienna, Austria; Instituto Butantan, BRAZIL

## Abstract

The objective of the present study was to assess safety and immune responses in gilts after intradermal application of Porcilis^®^ PRRS in two different application sites under field conditions. Forty-four gilts were allocated to one of three groups: Gilts of group 1 (*n* = 10) served as non-vaccinated controls, gilts of group 2 (*n* = 17) were vaccinated intradermally in the neck and gilts of group 3 (*n* = 17) received an intradermal vaccination in the perianal region. Clinical observations, local injection site reactions and histopathologic examination of the injection site were used for safety assessments. Frequency and degree of clinical signs were not significantly different between all three groups. Minor local reactions for both vaccination groups were observed; however, at 6, 7, 8, 9 and 15 days post-vaccination (dpv), the mean injection site reaction score was significantly lower in pigs vaccinated in the perianal region. In histopathologic examination, an extended inflammatory dimension was observed more frequently in pigs vaccinated in the neck. Blood samples were analyzed to quantify the post-vaccination humoral (ELISA and virus neutralization test) and cellular (IFN-γ ELISPOT) immune responses. PRRSV-specific antibodies were present in the serum of all vaccinated animals from 14 dpv onwards, whereas all control pigs remained negative throughout the study. Neutralizing antibody titers were significantly higher in pigs vaccinated in the perianal region at 28 dpv. At 14, 21 and 28 dpv, PRRSV-specific IFN-γ secreting cells were significantly increased in both vaccination groups compared to non-vaccinated gilts. Analysis of mean numbers of PRRSV-specific IFN-γ secreting cells did not result in statistically significant differences between both vaccination groups. The results of this study indicate that the perianal region is a safe alternative application site for intradermal vaccination of gilts with Porcilis PRRS. Furthermore, the intradermal application of Porcilis PRRS induced humoral and cellular immune responses independent of the administration site.

## Introduction

The development of needle free injection systems dates back to the 1930s [1]. These devices have been applied in human medicine for delivering insulin, anesthetics, growth hormones and vaccines [2–5]. The skin, as a highly effective component of the immune system, is an attractive target for vaccination due to its high density of immunocompetent cells such as Langerhans cells and dermal dendritic cells that specialize in antigen uptake followed by antigen presentation [6]. During the last decade, intradermal vaccination has also gained increasing interest in veterinary medicine. The needle-free intradermal route of antigen administration represents a less-invasive and less-painful alternative to conventional subcutaneous or intramuscular injections using a needle. Next to animal welfare improvement, additional merits of intradermal vaccination are its dose sparing capacity, reduction of iatrogenic transmission of pathogens, elimination of the risk of inadvertent needle stick injuries and improved meat quality due to the lack of needle-induced injection site lesions [7–9]. According to several investigations pigs have a high prevalence of injection site associated carcass defects [10]. Condemnations of carcasses are not only attributed to broken needles but also to abscesses and muscle damage. Currently, several commercially available vaccines against swine-relevant pathogens (i.e. *Mycoplasma (M*.*) hyopneumoniae*, porcine reproductive and respiratory syndrome virus (PRRSV), porcine circovirus 2, and pseudorabies virus) are licensed for the intradermal needle-free delivery route.

PRRSV is considered to be one of the major pathogens in pigs and causes substantial economic impact on the swine industry worldwide. Different strategies (i.e. management, test and removal, vaccination) have been implemented in PRRSV-infected herds to control the disease. A key component to reduce PRRSV-related reproductive and respiratory disorders is vaccination. Modified live virus vaccines have proven to be efficacious in reducing disease occurrence and severity, as well as duration of viremia and virus shedding [11–13]. Several studies that compare intradermal and intramuscular vaccination of PRRSV modified live virus suggest that intradermal administration of PRRSV vaccines is sufficient to trigger specific humoral and cellular immune response similar or even superior to intramuscular vaccination [13–15]. Porcilis^®^ PRRS is approved in the EU for intradermal application in the neck or along the muscle of the back using an intradermal device. An alternate application site, like the perianal region has the potential to increase impact on animal welfare and increase ease of access for the vaccinator, particularly in sows. However, data on vaccine safety and the resulting immune response for this application site is limited so far. Therefore, the present study was designed to assess and compare these parameters after intradermal application of Porcilis PRRS in the neck and the perianal region of gilts in a commercial farm setting.

## Materials and methods

### Study design

An empty pig facility was rented in order to conduct the study. At -7 dpv a total of 44 Danish landrace gilts, purchased from a high health herd, known to be free of PRRSV, *M*. *hyopneumonia*e and *Actinobacillus pleuropneumoniae*, confirmed by an actual screening shortly before study initiation, were included in the study. Gilts were individually ear tagged and were randomly allocated to one of three groups (groups 1, 2 and 3). After one week of acclimatization (0 dpv), gilts of group 2 (*n* = 17) were vaccinated intradermally (i.d.) in the neck using a live attenuated PRRS genotype 1 virus vaccine (Porcilis PRRS, MSD Animal Health, Germany) dissolved in Diluvac Forte, according to the manufacturer´s instructions (0.2 ml). For gilts of group 3 (*n* = 17), one dose of Porcilis PRRS was administered i.d. (0.2 ml) in the perianal region (off-label injection site). Intradermal vaccination was done with a needle free intradermal device (IDAL). The IDAL injector is a battery powered jet injector, equipped with a bottle holder completed with a spike, in which a vial of vaccine or rinsing fluid is fitted. Vaccination takes place using the injection head, which is fitted with a mechanical safety cylinder. The device is capable of delivering a “jet stream” of vaccine (0.2 ml) through the epidermal layers of the skin. For this purpose the device gives an initial peak force of 2.0–4.2 N to penetrate the skin followed by a vaccine delivery phase with the force decreasing over time and a drop-off phase where the force goes to zero (“force curve”). Gilts of group 1 (*n* = 10) remained unvaccinated and served as negative control group. Vaccinated pigs (group 2, 3) and pigs from the control group were housed in different barns with separate air spaces to prevent transmission of vaccine virus to control pigs. Clothing, footwear and gloves were changed between rooms and materials needed for sampling and rectal temperature monitoring were provided separately for each room. Animals in both barns were kept under similar conditions in terms of climate, ventilation, temperature and air humidity. All gilts were group-housed in groups of 17 (group 2, 3) or 10 (group 1) animals on partially slatted floor and space allowance of 2.5 m^2^/gilt. The gilts had permanent free access to drinking water and a chain and plastic ball combination was provided as environmental enrichment material. Fresh air entered through the ceiling and was discharged via a fan in the back of the unit. Artificial light was on from 6 AM to 10 PM. Gilts were fed twice a day with a standard diet for pregnant sows (135 g crude protein, 30 g crude fat, 62 g crude fiber, 54 g crude ash, 12.1 metabolizable energy per kg food) obtaining 2.5 kg feed daily.

General health of all gilts was monitored daily for a period of 28 dpv. For safety assessments, an individual examination of clinical signs was performed in vaccinated gilts of groups 2 and 3 one day before vaccination, 4h after vaccination and subsequently daily until 15 dpv. Clinical observation included an assessment of general health, appetite, body condition score, behavior, respiratory signs and digestion. The rectal temperature was measured with a digital thermometer on the day before and at the time of vaccination, 4 h later and on the consecutive four days. Injection sites of gilts from groups 2 and 3 were monitored for presence or absence of reactions including redness, swelling, changes in consistency, necrosis and pain during palpation at day 0 prior to vaccination, 4h post-vaccination and subsequently daily until 28 dpv by the same observer. Injection site reaction (ISR) parameters (Table 1) and clinical examination were evaluated according to a scoring system. Scores were added up and both, a mean cumulative general clinical score and ISR score were calculated for each day. Blood samples were collected by puncture of the jugular vein at -7, 0, 7, 14, 21 and 28 dpv and analyzed for virus-specific antibodies. At 0 dpv, prior to vaccination blood samples of all gilts were analyzed by quantitative reverse transcriptase PCR (qRT-PCR) as previously described by Kleiboeker et al. [16] to exclude circulation of field virus. At 7 dpv, samples from gilts of group 1 were investigated by qRT-PCR to exclude transmission of vaccine virus to the control group. Additionally, ten pigs per group (n = 30) were randomly selected and heparin stabilized blood samples were collected at 0, 7, 14, 21 and 28 dpv. Peripheral blood mononuclear cells (PBMCs) were isolated from these blood samples and used for IFN-γ ELISPOT assays. In addition, the ten randomly selected blood samples from both vaccination groups (groups 2 and 3; *n* = 20) were investigated for the presence of PRRSV-specific neutralizing antibodies (nAbs) at 28 dpv. At 28 dpv, pigs were euthanized by intravenous administration of pentobarbital (Release^®^, WDT- Wirtschaftsgenossenschaft Deutscher Tierärzte eG, 45 mg/kg body weight). Gilts from groups 2 and 3 were submitted for necropsy with subsequent microscopic examinations of the application site. Furthermore, the contralateral non-treated site of each animal served as control tissue. All procedures were reviewed and approved by the ethics committee of the District Government of Upper Bavaria under approval code Az.: 55.2-1-54-2532.0-43-14).

**Table 1:** Local injection site reaction score.

Parameter	Extent	Score
**Redness**	No	0
	Slight	1
	Severe	2
**Size of redness**	0.00–0.50 cm	0
	0.60–2.00 cm	1
	> 2.00 cm	2
**Swelling**	No	0
	Slight	1
	Severe	2
**Pain during palpation**	No	0
	Slight	1
	Severe	2
**Necrosis/ulceration**	No	0
	Slight	1
	Severe	2
**Induration**	No	0
	Firm	1
	Hard	2
**Maximum score**		**12**

### Laboratory analysis

#### Serology

Sera were analyzed for the presence of PRRSV specific antibodies by means of a commercial ELISA (IDEXX PRRS X3 Ab Test^®^, IDEXX Laboratories) using the immunoassay analyser ThunderBolt™ (Goldstandard Diagnostics U.S.A.) at the laboratory of the Clinic for Swine, Oberschleissheim, Germany. Results were expressed as sample to positive control (S/P) optical density ratios. According to the manufacturer, samples with S/P ≥ 0.4 were considered positive.

#### Virus neutralization tests to determine PRRSV-specific nAbs

The nAbs from the sera of the vaccinated animals were determined with a fluorescence-based neutralization assay (FBN-assay) on MA104 cells. Prior to the assay, a defined virus stock solution of Porcilis PRRS was cultivated for 3 passages on MA104 cells and titrated. For the neutralization assay, MA104 cells (5 x 10^3^ cells/well, 96-well flat bottom, Greiner Bio One, Frickenhausen, Germany) were cultivated for 2 days in Dulbecco´s Modified Eagle Medium (DMEM) (PAN Biotech, Aidenbach, Germany), 10% (v/v) heat-inactivated fetal calf serum (FCS, PAN, Pasching, Austria), 100 IU/mL penicillin and 0.1 mg/mL Streptomycin (both from PAN Biotech) until a 90% confluent cell monolayer was visible. Sera derived from the animal experiment were diluted 1:2 with cell culture medium (DMEM with supplements) and titrated in additional seven log2 steps (up to a dilution of 1:256). Thereafter a PRRSV-stock solution (plaque titer 1.28 x 10^6^ plaque forming units (PFU)/mL) was diluted 1:200 with DMEM without FCS to a concentration of 6.4 x 10^3^ PFU/mL and 100 μL were added to the serum dilutions and incubated for 45 min at room temperature (RT) (resulting in serum dilutions of 1:4 to 1:512). Thereafter MA104 cells were infected with 100 μL of the pre-incubated PRRSV serum solutions (multiplicity of infection [MOI] of 0.5). After 1h incubation at 34°C, 100 μL of DMEM (4% FCS) was added and the cells incubated for additional 24h at 37°C.

Thereafter, cells were fixed with 4% paraformaldehyde (100 μL/well, 20 min RT, Merck, Darmstadt, Germany), permeabilized with 0.1% (w/v) saponin (100 μL/well in phosphate buffered saline (PBS), 10 min RT, Sigma-Aldrich) and incubated with a monoclonal antibody against PRRSV-nucleoprotein (clone P10/b1, IgG1, [17], 50-μL cell culture supernatant, 45 min at RT), followed by two washing steps with PBS and an additional incubation step with goat-anti mouse IgG1-Alexa488 (Thermo Fisher Scientific, Waltham, MA, 50 μL, 1:200, 30 min RT) and two washing steps. Analyses were performed with a fluorometer (Spectrafluor, Tecan, Crailsheim, Germany) for the detection of N-protein-specific relative fluorescence intensities (RFIs). RFIs of the virus control (without nAbs) ranged between 7,000 and 9,000 units and RFIs of the negative control (non-infected) showed around 1000 RFIs. Due to endpoint titrations of the respective sera, nAb titers were determined when RFI values of the serum dilution reached the RFI level of the untreated virus control.

#### ELISPOT assay to quantify PRRSV-specific IFN-γ-secreting cells

The numbers of PRRSV-specific IFN-γ secreting cells (IFN-γ-SCs) were determined in PBMCs. PBMCs were isolated from heparinized blood samples by density gradient centrifugation (Pancoll human; density, 1.077 g/ml; PAN Biotech) as described elsewhere [18]. The IFN-γ ELISPOT assay was performed as previously described [19]. In brief, 96-well plates (MSIPS4510, Merck Millipore, Darmstadt, Germany) were coated over night with a monoclonal antibody specific for porcine IFN-γ (Clone pIFN-γ, Mabtech, Nacka Strand, Sweden), adjusted to 10 μg/mL. After washing, freshly isolated PBMCs were added to wells (3 × 10^5^ PBMCs per well) in RPMI 1640 with stable glutamine (PAN Biotech) supplemented with 10% (v/v) heat inactivated fetal calf serum (FCS, PAN Biotech), 100 IU/mL penicillin and 0.1 mg/mL streptomycin (PAN Biotech). Cells were stimulated with Porcilis^®^ PRRS vaccine reconstituted in cell culture medium and adjusted to MOI = 1. Cells cultivated in medium only served as negative control. All samples were analyzed in triplicates. After 24 h, PBMCs were discarded and plates incubated with a second IFN-γ-specific biotinylated antibody (clone PAN, Mabtech, 1 μg/mL) followed by incubation with streptavidin alkaline phosphatase (Roche, Mannheim, Germany). Finally, 5-bromo-4-chloro-3-indolyl phosphate/nitro blue tetrazolium substrate (Sigma-Aldrich, Vienna, Austria) was added for spot development. Spots were enumerated in a camera-based automated counting system (AID, Straßberg, Germany). Frequencies of IFN-γ SCs were calculated by subtracting spot numbers of medium-incubated cultures from spot numbers detected after PRRSV vaccine stimulation.

#### Histopathologic examination

After fixation of tissue samples in 4% buffered formaldehyde solution, paraffin embedding and slicing was performed; 4–5 μm thick slices were stained by hematoxylin and eosin (HE) and underwent histopathologic evaluation via optical microscopy by two independent investigators blinded to the treatments. Furthermore, immunohistochemical staining for T and B cells was performed using antibodies for CD3 (Polyclonal Rabbit Anti Human CD3, Dako, Glostrup, Denmark) or CD20 (Epitope Specific Rabbit Antibody, Dunn Labortechnik GmbH, Asbach, Germany) and CD79a (Mouse Anti-Human CD79a, Linaris Biologische Produkte GmbH, Dossenheim, Germany) respectively. Scoring of inflammation was performed in the following manner: (-) no, (+) mild, (++) moderate and (+++) strong inflammatory reaction. Furthermore, lesion dimension was estimated and the depth and expansion of invading inflammatory cells in dermal structures was recorded. The extension of the lesion was estimated in comparison to the whole slice given as percentages (1–5%, 6–10%, 11–15%, 16-20%, 21–25%, >25%).

### Statistical analysis

Sample size calculation was based on Mann-Whitney-U test with α = 0.025 and β = 0.20 using a case control ratio of 4. Adding a drop out, it revealed n = 17 for both vaccine groups and n = 10 for the control group based on PRRSV-specific IgG antibody response as primary variable. Clinical examination score, local ISR score, rectal temperature, PRRSV-specific IgG antibodies, IFN-γ producing cells in ELISPOT and PRRSV-nAb titers were compared between study groups by the Mann-Whitney-U test after testing for normal distribution of data. Persistence of local ISR were evaluated in contingency tables with Pearson`s chi-square tests. Spearman rank correlation coefficient was used to determine a correlation between IFN-γ producing cells and nAb titers. Data were analyzed using IBM SPSS Statistics 22, SPSS Inc., IL, USA. Significance level was 5% with a 95% confidence interval.

## Results

### Safety of intradermal vaccination

To assess the safety of an intradermal vaccination of Porcilis PRRS in the perianal region, local and systemic data were collected from all gilts until 28 dpv. During the observation period coughing was recorded in one control gilt. Slight tachypnea occurred in both vaccination groups (group 2: 3 animals, group 3: 4 animals). One animal vaccinated in the neck (group 2) had reduced feed intake and impaired general health due to lameness of the hind limb. No significant differences were found in mean clinical scores between both vaccination groups and between vaccinated animals and animals of the control group (data not shown). In both vaccinated groups the mean rectal temperature remained within physiological range and did not differ significantly between groups (data not shown).

Local injection site reactions, described as redness and papule with a size of up to 4 mm in diameter, were recognized in all gilts of both vaccination groups immediately after intradermal vaccination. The mean total ISR score is displayed in Table 2. At the individual pig level a maximum score of 7 out of 12 scoring points was observed in one gilt of group 2 at 24 and 25 dpv and 9 points in one gilt of group 3 at 16 and 17 dpv. Regarding individual parameters, the mean diameter of redness was significantly higher in pigs of group 2 (6 dpv: 1.42 cm; 7 dpv: 1.71 cm; 15 dpv: 1.43 cm) compared to pigs of group 3 (6 dpv: 0.71 cm; 7 dpv: 0.86 cm; 15 dpv: 0.55 cm) at 6, 7 and 15 dpv. In gilts that received Porcilis PRRS in the neck the maximum diameter of redness was 2.77 cm (one gilt, 7 dpv), whereas a maximum diameter of redness of 1.64 cm (one gilt, 4 dpv) was observed in gilts that were vaccinated in the perianal region. At 6, 7, 9 and 14 dpv, more severe swelling was observed in pigs of group 2 (average score for swelling: 6 dpv: 0.53, 7 dpv: 0.59; 9 dpv: 0.41; 14 dpv: 0.41) compared to pigs of group 3 (6 dpv: 0.12; 7 dpv: 0.06; 9 dpv: 0; 14 dpv). The remaining parameters (pain during palpation, necrosis, induration and degree of redness) did not differ significantly between both vaccination groups (data not shown). At day 28, the number of pigs with ISR did not differ significantly between both vaccination groups (group 2: 14/17; group 3: 11/17).

**Table 2:** Comparison of overall local injection site reaction score of both vaccination groups during the study period.

study day	score of group 3 (perianal; n = 17)				score of group 2 (neck; n = 17)				p value
	min^a^	max^b^	mean^c^	SD^d^	min^a^	max^b^	mean^c^	SD^d^	
**d-1**	0	0	0.00	0.00	0	0	0.00	0.00	1.000
**d0**	0	0	0.00	0.00	0	0	0.00	0.00	1.000
**d0+4**	2	3	2.76	0.44	1	3	2.88	0.49	0.433
**d1**	1	3	2.71	0.69	1	4	2.71	0.85	0.973
**d2**	1	4	2.53	0.94	1	4	2.76	0.83	0.474
**d3**	0	4	2.29	0.99	0	4	2.71	1.36	0.099
**d4**	1	4	2.59	0.71	0	5	2.65	1.41	0.413
**d5**	1	4	2.59	0.71	0	5	2.76	1.56	0.357
**d6**	1	4	2.65	0.86	0	5	3.59	1.33	0.013*
**d7**	0	4	2.76	0.90	0	6	3.88	1.41	0.005*
**d8**	0	3	2.41	1.06	0	6	3.47	1.37	0.018*
**d9**	0	3	2.35	1.06	0	6	3.18	1.47	0.034*
**d10**	0	3	2.29	1.05	0	5	3.00	1.37	0.073
**d11**	0	5	2.47	1.23	0	6	3.18	1.59	0.057
**d12**	0	6	2.53	1.46	0	6	3.29	1.65	0.150
**d13**	0	6	2.47	1.46	0	6	3.35	1.66	0.079
**d14**	0	7	2.41	1.66	0	6	3.35	1.66	0.057
**d15**	0	7	2.29	1.72	0	6	3.24	1.52	0.038*
**d16**	0	9	2.59	2.12	0	5	3.35	1.58	0.067
**d17**	0	9	2.65	2.32	0	5	3.18	1.59	0.160
**d18**	0	8	2.71	2.17	0	6	2.88	1.45	0.540
**d19**	0	8	2.65	2.15	0	6	3.00	1.54	0.322
**d20**	0	7	2.59	2.00	0	6	2.94	1.52	0.394
**d21**	0	6	2.24	1.99	0	5	2.76	1.48	0.306
**d22**	0	4	2.06	1.43	0	5	2.53	1.28	0.394
**d23**	0	4	2.06	1.43	0	5	2.59	1.33	0.306
**d24**	0	4	2.12	1.45	0	7	2.71	1.61	0.394
**d25**	0	4	2.00	1.46	0	7	2.65	1.69	0.339
**d26**	0	3	1.76	1.44	0	6	2.59	1.54	0.150
**d27**	0	3	1.71	1.40	0	6	2.47	1.74	0.182
**d28**	0	3	1.76	1.44	0	6	2.35	1.77	0.322

^a^Minimum (min)

^b^maximum (max)

^c^mean and

^d^standard deviation (SD) of injection site reaction score of both vaccination groups observed during the entire investigational period.

* indicate statistically significant differences between groups (p ≤ 0.05).

**Fig 1:**
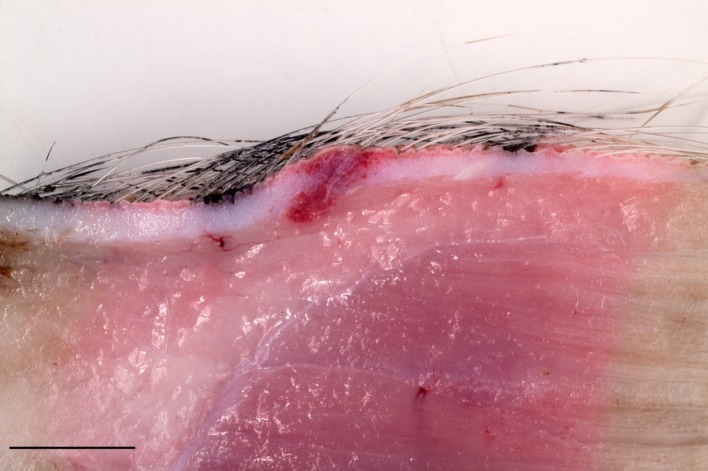
Representative micrograph for macroscopic examination of neck tissue 28 days after application of Porcilis PRRS. Micrograph shows focal red discoloration in the dermis with raising of the surface and slight protrusion of the lesion into the subcutaneous tissue. (Bar = 1cm).

**Fig 2:**
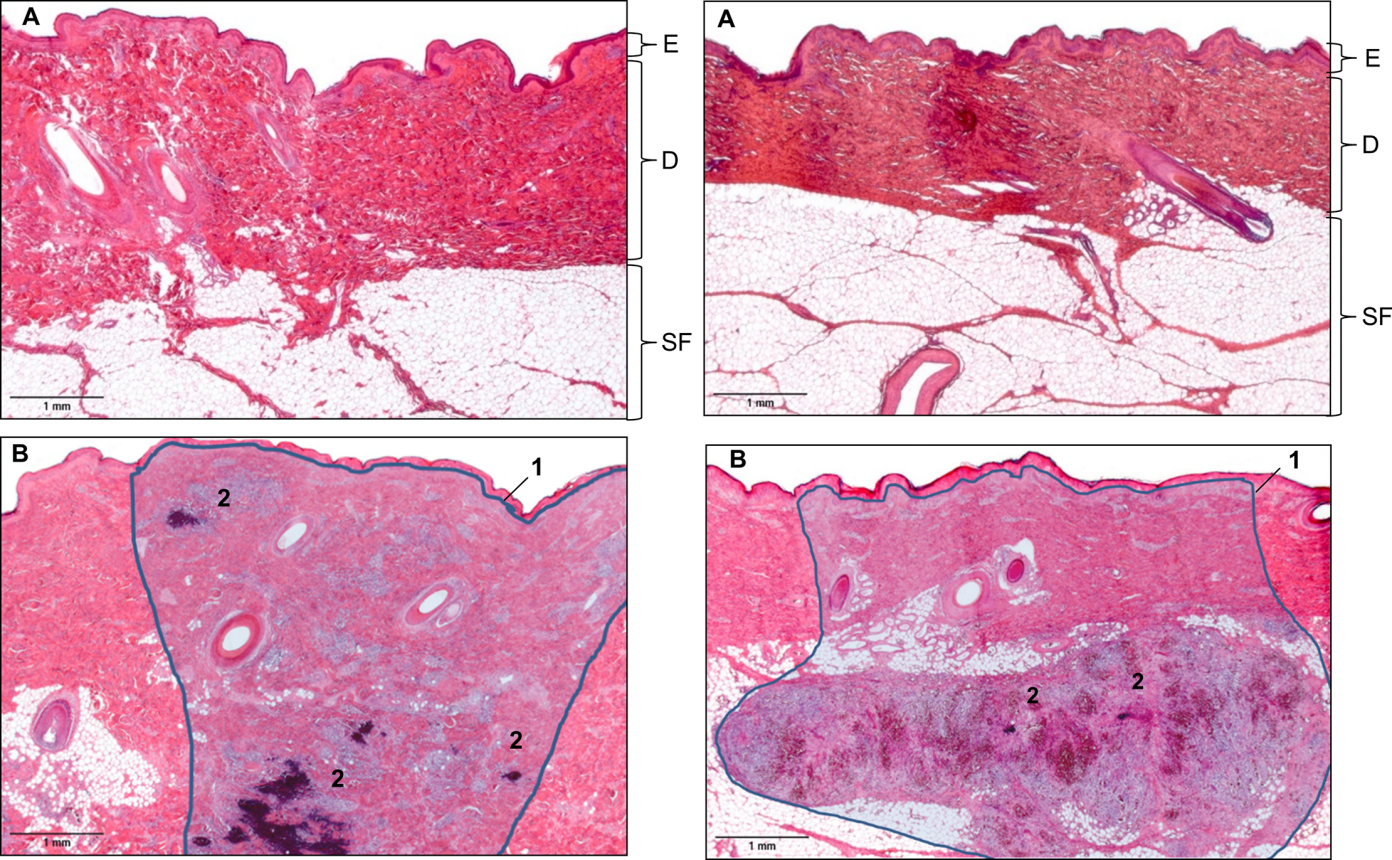
Histopathological changes of tissues samples of the neck and the perianal region 28 days after application of Porcilis PRRS. A) HE stained slides of control tissue (contralateral, non-treated site of neck [left] and perianal region [right]) without any inflammation. B) HE stained slides of the neck [left] and perianal application [right] sites: moderate inflammation with fibrosis and cellular infiltrates reaching the subcutaneous fat tissue. Abbreviations: E = Epidermis, D = Dermis, SF = Subcutaneous fat tissue; 1 = Inflammatory focus, 2 = Inflammatory cellular infiltration with multifocal necrosis and dystrophic calcification. Representative slides of the different treatment groups are shown.

Histopathologic examination revealed inflammatory reactions at the injection site in all treated animals, whereas none of the control sites had any inflammation. The injection site in all animals of both groups included one inflammatory focus of variable dimension with several spots of higher density of cellular infiltrates. Furthermore, foci of necrosis and dystrophic calcification were found in the majority of animals of both vaccination groups (Figs 1 and 2). Histopathologic findings were accentuated in the papillary layer of the dermis. Single cases (1 perianal and 7 neck injected animals) showed infiltrates reaching the subcutaneous fat tissue. The extension of the lesion differed significantly (p = 0.002) between both vaccination groups (median: group 2: 16–20%; group 3: 11–15%). Lymphocytes dominated in inflammatory lesions, representing both, T and B cells of similar distribution pattern in all animals. Furthermore, plasma cells and some neutrophils and macrophages were detected (data not shown). In six animals of group 2 and in four animals of group 3, multinucleated giant cells were found in the periphery of necrotic foci (data not shown). In addition, in all animals, fibrosis of the injection site was present.

### PRRSV immune responses

To exclude circulation of PRRS field virus as well as transmission of vaccine virus to the control group qRT-PCR was performed at 0 dpv in samples of all animals and at 7 dpv in samples of control animals. Blood samples from all gilts were negative for PRRSV RNA by qRT-PCR prior to vaccination and PRRSV RNA could not be detected in gilts of the control group at 7 dpv.

#### PRRSV-specific ELISA antibody response

**Fig 3:**
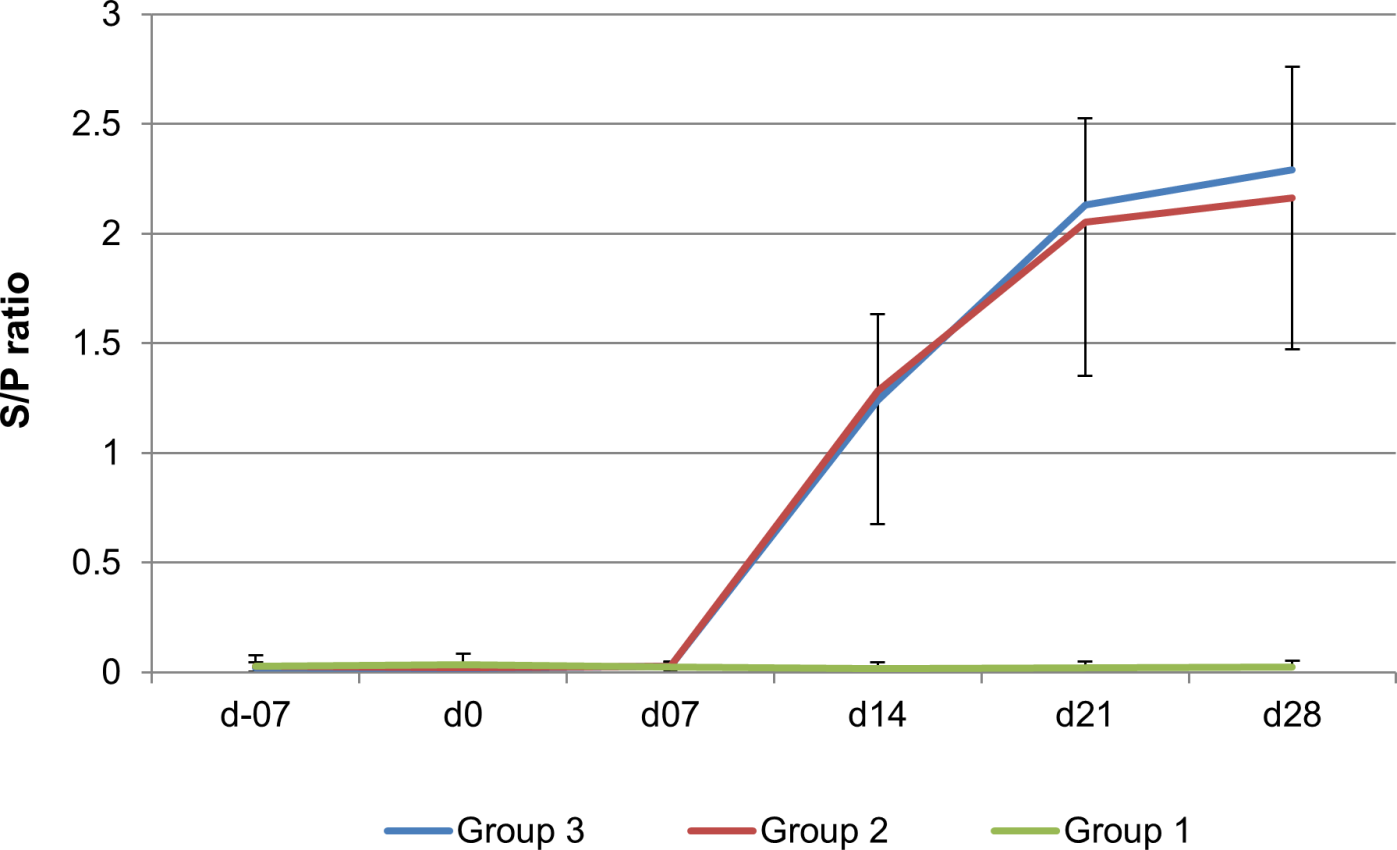
PRRSV-specific antibody titers (S/P ratios) by ELISA in the time course of the different groups. S/P ratio for non-vaccinated gilts (group 1), gilts that received intradermal PRRSV vaccination into the neck (group 2) and sows that received intradermal PRRSV vaccination into perianal region (group 3) are shown. Mean S/P ratios for the three different groups ± standard deviations are shown.

The humoral PRRSV immune response was evaluated in terms of PRRSV-specific antibodies in serum with a commercially available ELISA. Results are presented in Fig 3. Prior to vaccination, at -7 dpv all gilts were seronegative. Blood samples from unvaccinated gilts of the control group yielded negative results at all collection time points. Antibodies against PRRSV could not be detected at 0 dpv and 7 dpv in serum samples of vaccinated pigs (groups 2 and 3). However, at day 14 PRRSV-specific antibodies were present in serum of all vaccinated animals. All gilts from groups 2 and 3 remained seropositive during the rest of the study. No significant differences were observed in antibody S/P ratio’s between both vaccination groups.

#### PRRSV-specific nAbs

**Fig 4:**
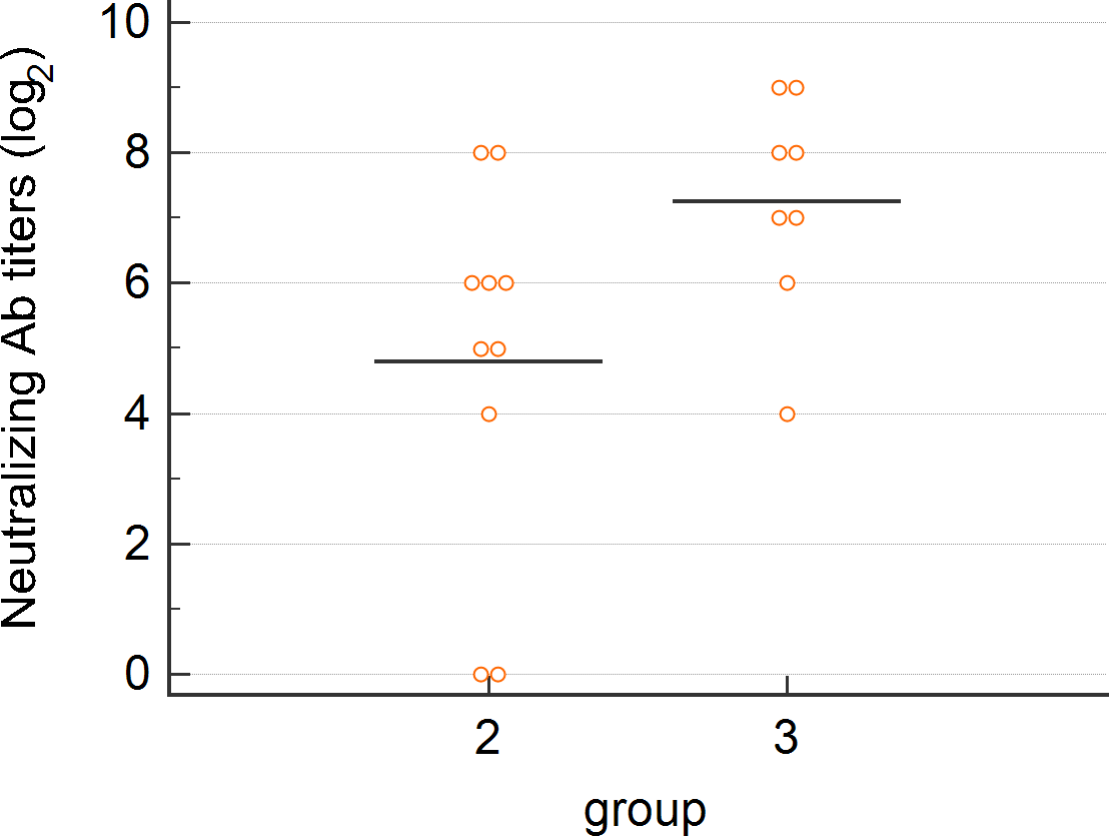
Comparison of PRRSV-nAb titers of vaccinated animals 28 dpv. Each symbol represents nAb titer of one individual animal vaccinated intradermally, either into the neck (group 2) or into the perianal region (group 3). Black bars represent mean values within each group.

To define nAb responses, virus nAb titers were determined by FBN assay at 28 dpv. NAbs were detected in 8/10 pigs vaccinated in the neck region with titers ranging from 1:16 to 1:256 (Fig 4). Only 2 gilts reached high titers of 1:256 while nAb titers of the remaining 6 gilts ranged between 1:16 and 1:64. Due to bacterial overgrowth during incubation, nAbs could not be evaluated in 2 gilts vaccinated in the perianal region. All 8 investigated gilts were positive for nAbs, with titers ranging between 1:16 and 1:512. Gilts vaccinated intradermally in the perianal region had significantly higher nAb titers than pigs vaccinated intradermally in the neck (p = 0.043). In contrast, PRRSV-specific nAbs were not detected in sera from non-vaccinated gilts (group 1).

#### Frequency of PRRSV-specific IFN-γ secreting cells

**Fig 5:**
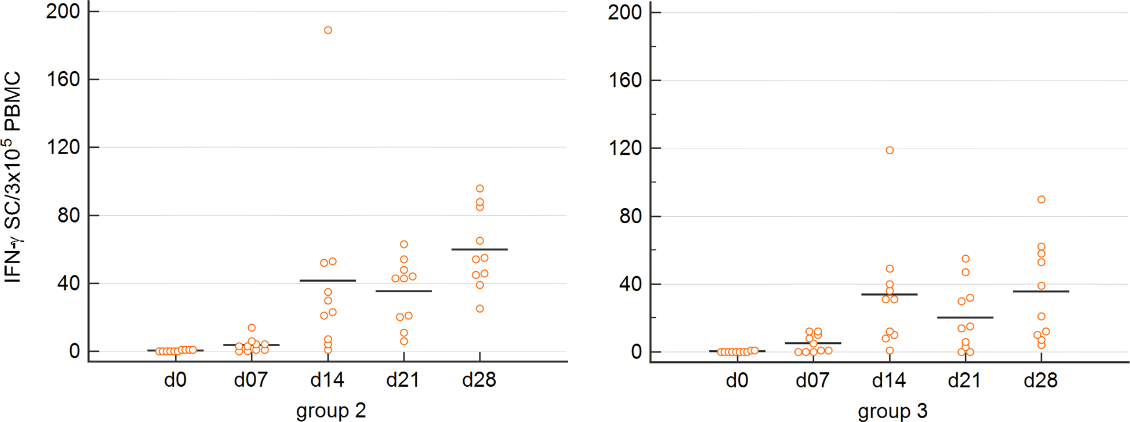
Frequency of PRRSV-specific IFN-γ producing PBMCs analyzed by ELISPOT. The consecutive graphs show the frequency of IFN-γ SCs in animals vaccinated intradermally into the neck (group 2) and animals vaccinated intradermally into the perianal region (group 3). Each symbol represents the frequency of IFN-γ producing cells of individual animals within 3 × 10^5^ PBMCs. Black bars represent mean values within each group per study day.

To further characterize the immunological response to PRRSV intradermal vaccination, PRRSV-specific IFN-γ SCs were measured. At 0 dpv, PRRSV-specific IFN-γ SCs could not be detected in animals from all three groups (Fig 5). At 7 dpv, low numbers of PRRSV-specific IFN-γ SCs were observed in both vaccination groups. The frequency of such cells increased further towards 14 dpv in both groups, although considerable animal to animal variations were observed. At 21 dpv, on average, a minor decrease of IFN-γ SCs was observed in both groups followed by a rise at 28 dpv. In contrast, animals from the control group showed no to a very low IFN-γ response with a maximum of 3 SCs/3 x 10^5^ PBMCs at all investigated time points (data not shown). At 7, 14, 21 and 28 dpv the number of IFN-γ SCs in both vaccination groups (group 2, 3) were significantly different as compared to the control group (group 1). The number of PRRSV-specific IFN-γ SCs tended to be higher in pigs vaccinated in the neck compared to gilts vaccinated in the perianal region. However, no significant differences between both vaccination groups were observed at any of the investigated time points (7, 14, 21, 28dpv) (p<0.05).

#### Association between nAb response and IFN-γ secreting cells

The frequency of PRRSV-specific IFN-γ SCs/3 x 10^5^ PBMCs was compared to the nAbs of individual animals. Interestingly, there were no detectable levels of nAbs in two animals 28 dpv despite their IFN-γ response. Comparison of PRRSV-nAb titers and IFN-γ SCs suggested that animals with low PRRSV-nAb titers had higher IFN-γ SCs than animals with high PRRSV-nAb titers. Hence, no positive correlation was observed between nAb titers and the number of IFN-γ SCs (p = 0.542).

## Discussion

Although several studies have shown that intradermal application of an attenuated PRRSV vaccine in the neck can efficiently induce a protective immune response [13, 14], up to date no studies are available investigating safety and efficacy of different application sites. As the neck is the most common site of mainly intramuscular injection of breeding stock an alternative application site could reduce stress for the animal and simplify the application process for the user. Therefore, the present study was designed to evaluate safety and humoral and cellular immune responses in gilts after intradermal vaccination with Porcilis PRRS in the perianal region. Since efficacy of intradermal vaccination of Porcilis PRRS in terms of clinical protection has been demonstrated in a previous study [13] no experimental infection was considered for the present study. Additionally, for ethical considerations regarding reduction of animals used in experiments an intramuscular group was not included in the study design as results from previous studies indicate that Porcilis^®^ PRRSV administered via the intradermal route can efficiently induce a protective immune response comparable or in some cases higher than the intramuscular route.[13–15, 20].

With respect to safety, no significant differences were found in frequency and degree of clinical signs between both vaccination groups and between vaccinated animals and control animals. In accordance with other studies, the intradermal application of Porcilis PRRS induced no systemic reactions, nor an increase in rectal temperature [14]. Local ISR were observed in both vaccination groups until 28 dpv. In contrast, in a recent study, ISR after intradermal application of Porcilis PRRS were resolved by 28 dpv [21]. However, in consistency with other studies, using the intradermal route of vaccination, the observed ISR were of minor degree [20, 22, 23]. In the present study, alterations of the skin were significantly more severe on 6, 7, 8, 9 and 15 dpv in pigs vaccinated intradermally in the neck compared to pigs vaccinated in the perianal region. As local reactions at the injection site could be associated with pain, vaccination in the perianal region might improve animal welfare. The overall degree and type of inflammation detected by histopathological examination was comparable between both vaccination groups. However, the extent of the inflammation was higher in the pigs vaccinated in the neck compared to perianal injected pigs. The differences in the extent of inflammation between both vaccination groups reflects the tendency of higher macroscopic injection site scores observed in pigs vaccinated in the neck. According to the literature, the penetration depth might vary between different application sites due to skin variability (i.e. skin thickness, skin surface, hair density) [24]. Therefore, the site of application seems to have an effect on grossly visible reaction levels and lesion extension at the injection site.

Vaccination with the intradermally administered vaccine evoked a PRRSV-specific antibody response measured by ELISA in all (34/34) animals from both vaccination groups on 14 dpv. Despite the fact that these results are in agreement with previous studies [14], it is well accepted that the concentration of serum antibodies measured by ELISA cannot be regarded as correlates of protective immunity against PRRSV [25–27]. In contrast, the cell mediated immune response in terms of frequency of IFN-γ SCs has been widely used to evaluate vaccine efficacy [13, 28–32]. According to Zuckermann et al. [29] the IFN-γ response can be used as indicator of protective immunity and also Martelli et al. [13] demonstrated a clear association between clinical protection and the kinetics of the cell mediated immune response. Similarly, Lowe et al. [30] showed that the number of IFN-γ producing cells measured by ELISPOT was correlated with protection against PRRSV in three of four commercial herds experiencing outbreaks of PRRSV and results from Diaz et al. [33] indicate a strong involvement of IFN-γ in the development of immunity against PRRSV. Also, Charerntantanakul et al. [28] showed that expression of IFN-γ by several T-cell subsets correlated with reduced lung lesions and viremia. Due to these previous findings, a challenge infection was not performed in our study, instead, we considered the simultaneous analysis of PRRSV-specific IFN-γ producing lymphocytes and nAbs (see also below) as adequate read-outs to evaluate vaccine efficacy. In previous studies intradermal administration of PRRSV vaccines provoked an IFN-γ response similar or even superior to the application via the intramuscular route [13, 15]. In the present study, a minor increase in IFN-γ SCs was detected in some pigs from both vaccination groups as early as 7 dpv. On 14 dpv, IFN-γ-SCs were evident in the vast majority of animals from both vaccination groups. In contrast, a different study investigating the intradermal route of PRRSV vaccination found a lower percentage of animals with PRRSV-specific IFN-γ-SCs at 14 dpv [13]. After 14 dpv, a further increase of IFN-γ SCs was observed in the present study. However, individual animals reached a maximum level at day 14, followed by a decline of IFN-γ SCs at 21 dpv and a subsequent increase at the end of the observational period (28 dpv). This is in contrast to other studies describing a gradual increase in the frequency of IFN-γ SCs [32]. However, biphasic IFN-γ SCs kinetics have also been observed by others [33, 34] and might be attributed to the highly variable IFN-γ SCs response among individual animals that has been observed in our study and also by others [34, 35]. According to Ferrari et al. [15], pig to pig variation was greater in pigs vaccinated intradermally compared to pigs vaccinated intramuscularly. Results from a recent study suggest that inter-individual variability in markers of PRRSV vaccine-induced immunity, including nAb levels and IFN-γ responses, may be regulated by host genetic factors [36]. Nevertheless, the mean number of IFN-γ SCs did not differ significantly between both vaccination groups. The intensity of the IFN-γ response observed in our study is consistent with the results obtained in previous studies [13, 15, 29]. In general, the frequency of IFN-γ SCs is reported to be low after vaccination or infection [13, 15, 29]. However, results from Klinge et al. [37] indicated that inoculation with virulent PRRSV strains elicits a higher number of IFN-γ SCs than inoculation with a modified live virus vaccine. It has to be kept in mind though that a comparison of IFN-γ SCs between different studies has to be done with caution as results are influenced by inter-assay variability caused by differences in restimulation time, MOI, type of antigen used for restimulation, the particular PRRSV strain used for infection and re-stimulation, as well as the immune status and the age of the pig [37]. In the present study vaccine virus was used as the recall virus in order to perform a homologous re-stimulation. According to our experience and in-house data, IFN-γ SCs in vaccinated pigs are induced after re-stimulation with homologous vaccine virus as well as with heterologous field virus (data not published). These findings can be explained by data of Mokhtar et al. [38], who used a peptide library spanning the entire PRRSV proteome in order to analyze the antigen specificity of T cell responses. The authors could identify highly conserved epitopes present within different PRRSV isolates.

Although several authors have highlighted the importance of nAbs for protective immunity against PRRSV [26, 27, 39] the role of nAbs in clearance of viremia and protection against the disease has been discussed controversially [40–42]. Results from a previous study indicate that protection is dependent on the amount of nAbs. However, age dependent differences have to be considered since higher concentrations of nAbs are needed to obtain sterilizing immunity in young piglets than in sows [43]. According to previous reports attenuated PRRSV vaccines induce a non-detectable or low nAb response, particularly if pigs are only vaccinated once [29, 44–46]. Ferrari et al. [20] did not observe nAbs in pigs vaccinated intramuscularly or intradermally with a modified live PRRSV vaccine prior to challenge (0–35 dpv). In contrast, the majority of pigs in our study (16/18) had nAbs at 28 dpv. Additionally, it is worth noting that high titers of nAbs were detected in the present study. However, it has to be kept in mind that nAb titers from different studies cannot be directly compared due to several factors influencing the outcome, like e.g. the particular PRRSV strain, the conditions of virus culture, visualization method, etc. In the present study, gilts vaccinated intradermally in the perianal region had significantly higher PRRSV-nAb titers than pigs vaccinated intradermally in the neck. Interestingly, two animals of the neck group did not develop nAbs on 28 dpv despite having medium to high numbers of PRRSV-specific IFN-γ SCs. As nAbs are known to appear late after infection/vaccination, typically ≥ 28 days [32, 47], the lack of nAbs at 28 dpv might be attributed to their delayed development. It is fairly remarkable that in contrast to previous reports [20, 34], no association was observed between virus nAbs and IFN-γ-SCs levels under the condition of this study. This might be explained by the short investigational period of 28 days. However, it seems more likely that this observation was mainly attributed to the extreme variability of IFN-γ SCs response observed among individual animals.

## Conclusion

Several studies have been conducted comparing humoral and cellular immune responses induced by intramuscular and intradermal PRRSV vaccination. However, up to date investigations on the compatibility of different intradermal application sites are lacking. As the neck is routinely used for vaccination, an alternative application site is highly desirable to improve welfare of animals. Results from the present study strongly suggest that the perianal region could be a safe application site and that the intradermal application of Porcilis PRRS in the perianal region induces cellular and humoral immune responses similar to the application into the neck.

## Supporting information

S1 Dataset (XLSX)
